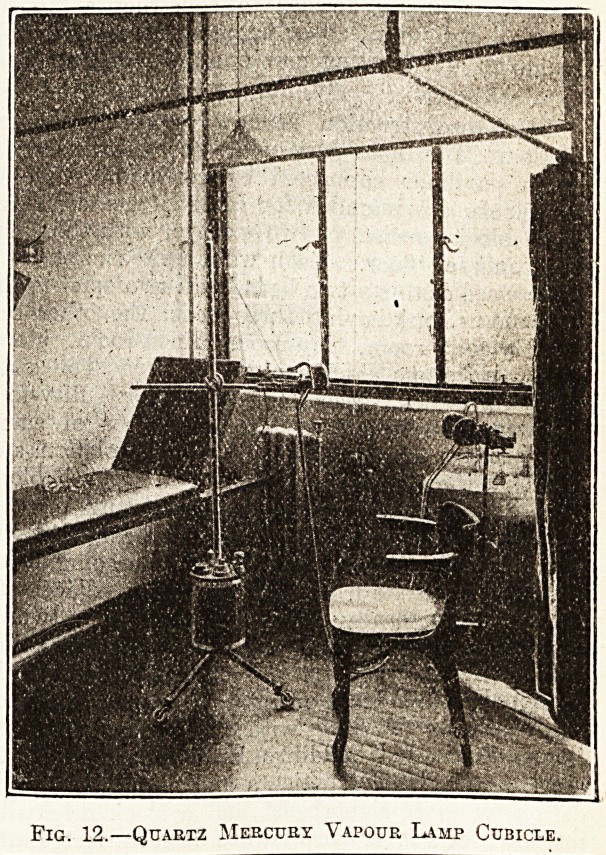# The Electrical and Radio-Therapeutic Department of the Cancer Hospital (Free), London

**Published:** 1914-08-08

**Authors:** Alfred M. Hooper

**Affiliations:** Assistant Secretary to the Hospital


### THE ELECTRICAL AND RADIO-THERAPEUTIC DEPARTMENT OF THE CANCER HOSPITAL (FREE), LONDON.

### II.—Details of Equipment and Cost.

By ALFRED M. HOOPER, Assistant Secretary to the Hospital.

Single-Impulse Induction Coil Outfit (plates 6 and 7).—This is contained in a large cabinet, half of which is used for the induction coil, Wehnelt interrupter, liquid condensers, oscilloscope tubes and valve tubes; the other half forms a protective cabinet for the operator, being lead lined and provided with lead-glass windows, contains the switchboard for controlling the Wehnelt and mercury brakes, and the single-impulse switch. The large coil of the apparatus will give a spark of 16 ins., with heavy currents for instantaneous work, and is wound in three parts so as to also give lighter currents for therapeutic purposes. Cost, £293.

*X*-ray Couch (plate 7).—This was specially designed by the director. Beneath the couch a Roentgen tube is fitted in such a manner that by the easy manipulation of handles the bulb is raised, lowered, or moved horizontally. Another tube is fitted above the couch for overhead work. The sides of the couch are lead lined for the protection of the operator. Cost, £57.

Reider Screen Examination Apparatus.—This apparatus is constructed in such a manner that the operator by moving the hanging screen at the same time moves the tube-box into the correct position. It is provided with a switch table which controls the current in the tube. Cost, £25.

Therapeutic outfit, with 12-inch spark induction coil, 7-inch dial milliamperemeter, oscilloscope and Lodge valve tubes, automatic cut-out arrangement, sparking pillars, cables, switchboard, centrifugal mercury interrupter, automatic clock switch, and an additional automatic regulator for controlling the vacuum at a distance for automatic and osmosis regulators. £65.

Therapeutic outfit as above, but fitted with Reiniger, Gilbert and Schall's apparatus for impulse interruption. £75 10s.

Cost of fitting up three *x*-ray cabinets with overhead tube-boxes on running rails, with universal movements, pastille holders, aluminium filters, and lead-glass applicators. £36.

Two electrolyte interrupters with 3 mm. platinum points for each therapeutic cubicle. £11 16s.

Universal electro-medical apparatus, earth free, with table and drawer for electrodes, cautery outfit with burners and ligature tubes, forehead lamp, ear and nose speculæ, laryngoscopes, cables and electrodes for galvanisation, faradisation, and ionisation, electrolysis outfit, Breuning's outfit, three œsophagus tubes, Schnee four-cell bath with porcelain baths and adjustable chair, four-cell bath commutator, chair with armrests and adjustable head clamp, nickel-plated. £84 8s.

Diathermy apparatus, with motor generator for connecting to supply, with foot contact with special electrode holder; electrolytic resistance, surgical electrodes, aseptic operation table and set of surface electrodes; high frequency for using table as high frequency treatment couch, etc. £91 10s.

High frequency apparatus for connecting the diathermy apparatus with adjustment, with rubber wheels, hot-wire ammeter, multiple point electrodes, single point electrode, condenser electrode set of vacuum electrodes, etc. £29 11s.

Intensifying screens, 15 inches by 12 inches and 12 inches by 10 inches. £10 18s.

Tubes.—Four; two air-cooled, two water-cooled, two radiator, two clover-leaf, one triple valve, two Oliver Lodge, one Holzknecht radiometer with colour scale, one Walter radiometer, one Benoist water radiometer, cassette for stereoscopic exposures for plates up to 16 by 20 inches, and large air-pump for air-cooled tubes. £85 12s. 6d.

Fig. 6.—Interior of Cabinet Containing Single-impulse *X*-ray Outfit.
Showing Induction Coil, Liquid Condensers, Wehnelt and Mercury Interrupters, Single-impulse Switch, Main Switchboard, and Forming Switch.

Opaque blinds, £81; curtains, rods, etc., for cubicles, £37; Teoplar pump with pressure pump, etc., £14 10s.; five couches, upholstered, length 5 feet 10 inches, £20; quartz mercury vapour lamp and extra applicators, £31 7s. 6d.

The hospital, starting with a modest purchase of £20, now possesses radium to the value of about £4,800; of this amount £1,000 has been presented (£500 by the "*Daily Express* Radium Fund" and the balance by a private donor). Sixty-five milligrammes is kept in solution for the purpose of

Fig. 7.—Radiographic Room.
Showing Exterior of Single-impulse Outfit with Cabinet, with Movable Regulating Switch, X-ray Couch, and Screening Apparatus.

Fig. 8.—One of the X-ray Cabinets.
Showing Apparatus with Coil Milliamperemeter, Switchboard, Mercury Interrupter, Rhythmical Apparatus, Clock Switch and Circuit-breaker on Exterior, Couch and Protective Tube Carrier on Inside.

Fig. 9.—Diathermy Cubicle.
Showing Apparatus, Surgical and Heating Electrodes, Foot Switch, and Operating Table.

obtaining emanations; the remainder is contained in platinum tubes, one of which contains 100 milligrammes, others 50 and smaller amounts. The department also possesses $5\frac{1}{2}$ grammes of actinium and mesothorium to the value of £20, the latter also a present.

The experimental work in connection with this department is carried on in a laboratory in the research institute. The room is situated in the basement and—as is highly important for the delicate apparatus used—it is very free from vibration. The equipment is very complete, with electroscopes, measuring appliances, photomicrographic apparatus, testing batteries, etc. The "Dolezalek" electrometer is mounted on a brick pillar and is enclosed in an earthed metal-lined case. An optical bench

Fig. 10.—High-frequency Cubicle.
Showing Apparatus, Couch, and Electrodes.

fitted with an electrometer and radium holder has a graduated steel rule 11 feet long. The testing battery gives 300 volts and consists of 200 Leclanche cells. Most of the apparatus was specially designed by the physicist. The work carried on in this laboratory includes the invention of various devices in connection with the application of radium and radium emanations which have proved of great value. By an arrangement of opaque blinds the room can be made absolutely dark.

The total cost of maintenance for the year 1913 amounted to £745, the principal items of expenditure being salaries and wages, £350; photographic plates, papers, etc., £140; new *x*-ray bulbs and re-charging, £87; fuel, £10. A sum of £30 was expended on providing outside blinds, and about £10 for timber for the construction of teak benches for the patients' waiting-hall.

Fig. 11.—Photographic Studio.
Showing Camera, Arc-lamp, Background, and Special Blinds for Daylight Illumination.

Fig. 12.—Quartz Mercury Vapour Lamp Cubicle.

[The illustrations are reproduced by kind permission of the Editor of *Archives of the Roentgen Ray*.]

## Figures and Tables

**Fig. 6. f1:**
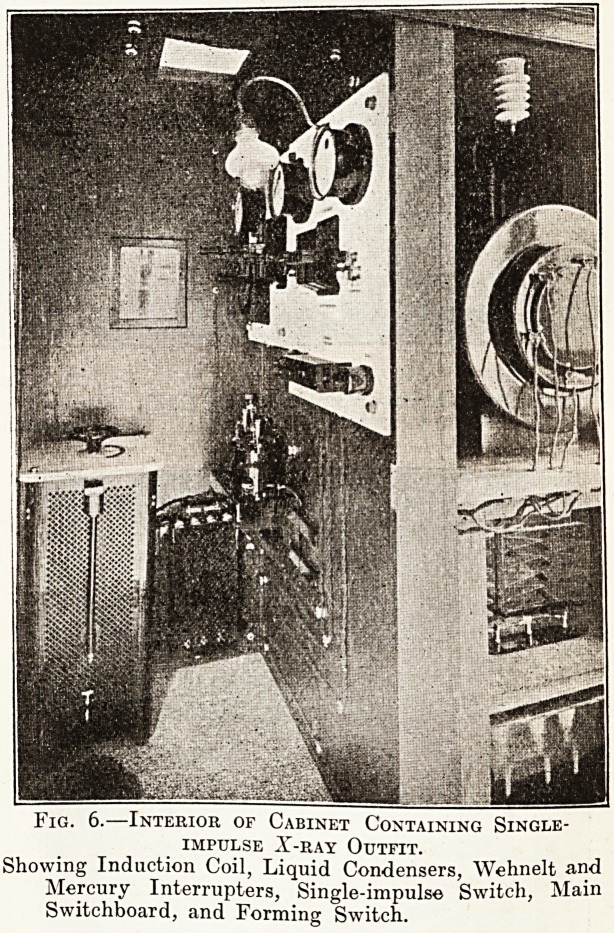


**Fig. 7. f2:**
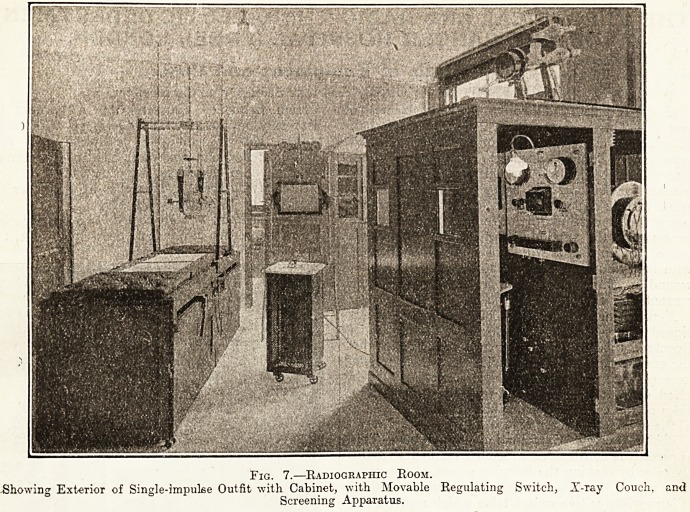


**Fig. 8. f3:**
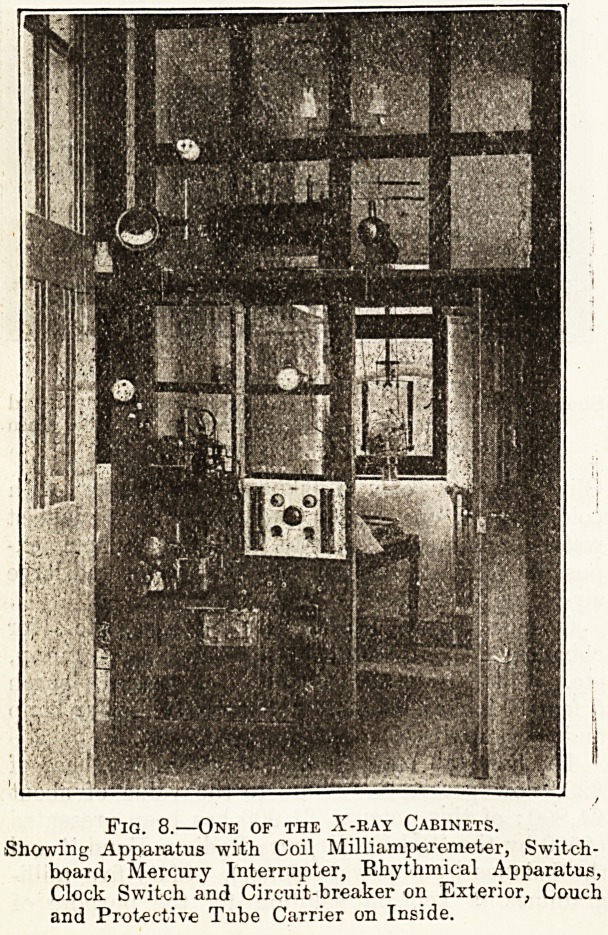


**Fig. 9. f4:**
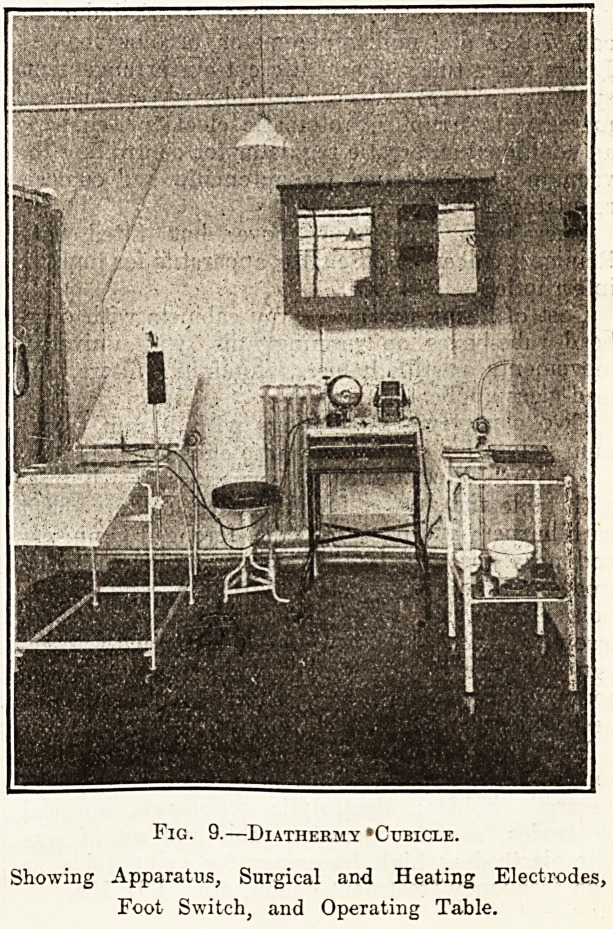


**Fig. 10. f5:**
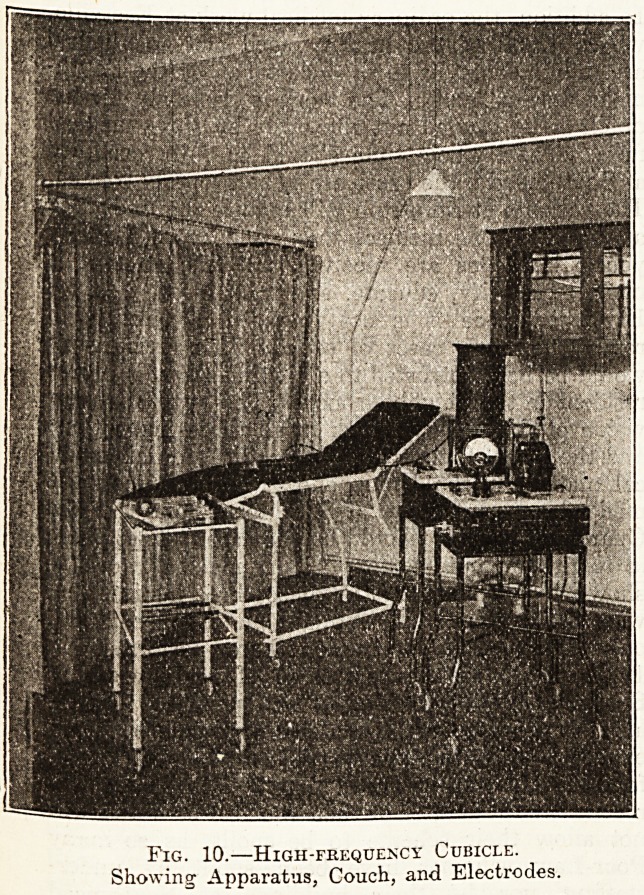


**Fig. 11. f6:**
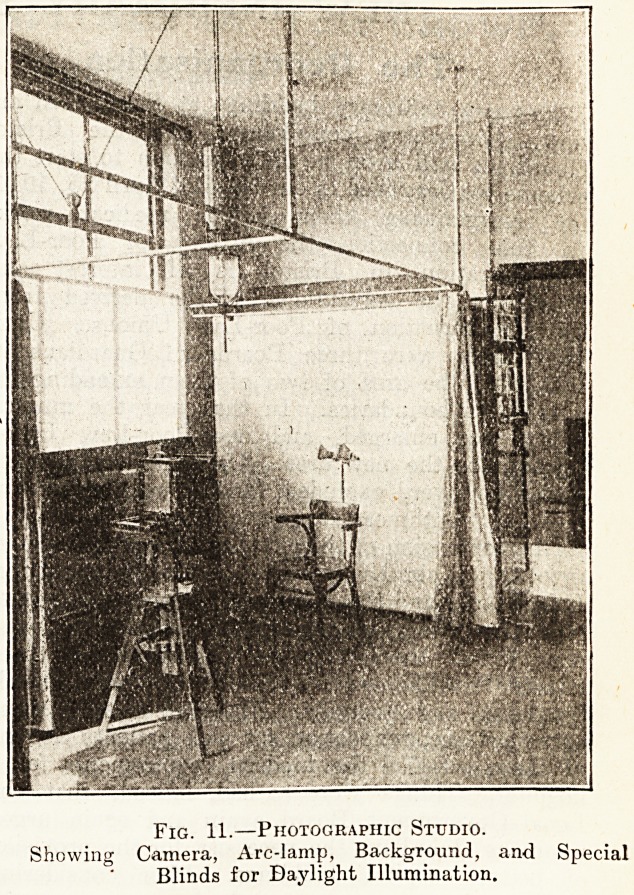


**Fig. 12. f7:**